# The role of Kisspeptin signaling in Oocyte maturation

**DOI:** 10.3389/fendo.2022.917464

**Published:** 2022-08-22

**Authors:** Saeed Masumi, Eun Bee Lee, Iman Dilower, Sameer Upadhyaya, V. Praveen Chakravarthi, Patrick E. Fields, M. A. Karim Rumi

**Affiliations:** Department of Pathology and Laboratory Medicine, University of Kansas Medical Center, Kansas City, KS, United States

**Keywords:** Ovarian follicles, kisspeptin, kisspeptin receptor, GnRH, gonadotropin, oocyte maturation, assisted reproductive technologies

## Abstract

Kisspeptins (KPs) secreted from the hypothalamic KP neurons act on KP receptors (KPRs) in gonadotropin (GPN) releasing hormone (GnRH) neurons to produce GnRH. GnRH acts on pituitary gonadotrophs to induce secretion of GPNs, namely follicle stimulating hormone (FSH) and luteinizing hormone (LH), which are essential for ovarian follicle development, oocyte maturation and ovulation. Thus, hypothalamic KPs regulate oocyte maturation indirectly through GPNs. KPs and KPRs are also expressed in the ovarian follicles across species. Recent studies demonstrated that intraovarian KPs also act directly on the KPRs expressed in oocytes to promote oocyte maturation and ovulation. In this review article, we have summarized published reports on the role of hypothalamic and ovarian KP-signaling in oocyte maturation. Gonadal steroid hormones regulate KP secretion from hypothalamic KP neurons, which in turn induces GPN secretion from the hypothalamic-pituitary (HP) axis. On the other hand, GPNs secreted from the HP axis act on the granulosa cells (GCs) and upregulate the expression of ovarian KPs. While KPs are expressed predominantly in the GCs, the KPRs are in the oocytes. Expression of KPs in the ovaries increases with the progression of the estrous cycle and peaks during the preovulatory GPN surge. Intrafollicular KP levels in the ovaries rise with the advancement of developmental stages. Moreover, loss of KPRs in oocytes in mice leads to failure of oocyte maturation and ovulation similar to that of premature ovarian insufficiency (POI). These findings suggest that GC-derived KPs may act on the KPRs in oocytes during their preovulatory maturation. In addition to the intraovarian role of KP-signaling in oocyte maturation, *in vivo*, a direct role of KP has been identified during *in vitro* maturation of sheep, porcine, and rat oocytes. KP-stimulation of rat oocytes, *in vitro*, resulted in Ca^2+^ release and activation of the mitogen-activated protein kinase, extracellular signal-regulated kinase 1 and 2. *In vitro* treatment of rat or porcine oocytes with KPs upregulated messenger RNA levels of the factors that favor oocyte maturation. In clinical trials, human KP-54 has also been administered successfully to patients undergoing assisted reproductive technologies (ARTs) for increasing oocyte maturation. Exogenous KPs can induce GPN secretion from hypothalamus; however, the possibility of direct KP action on the oocytes cannot be excluded. Understanding the direct *in vivo* and *in vitro* roles of KP-signaling in oocyte maturation will help in developing novel KP-based ARTs.

## Introduction

**Figure 1:**
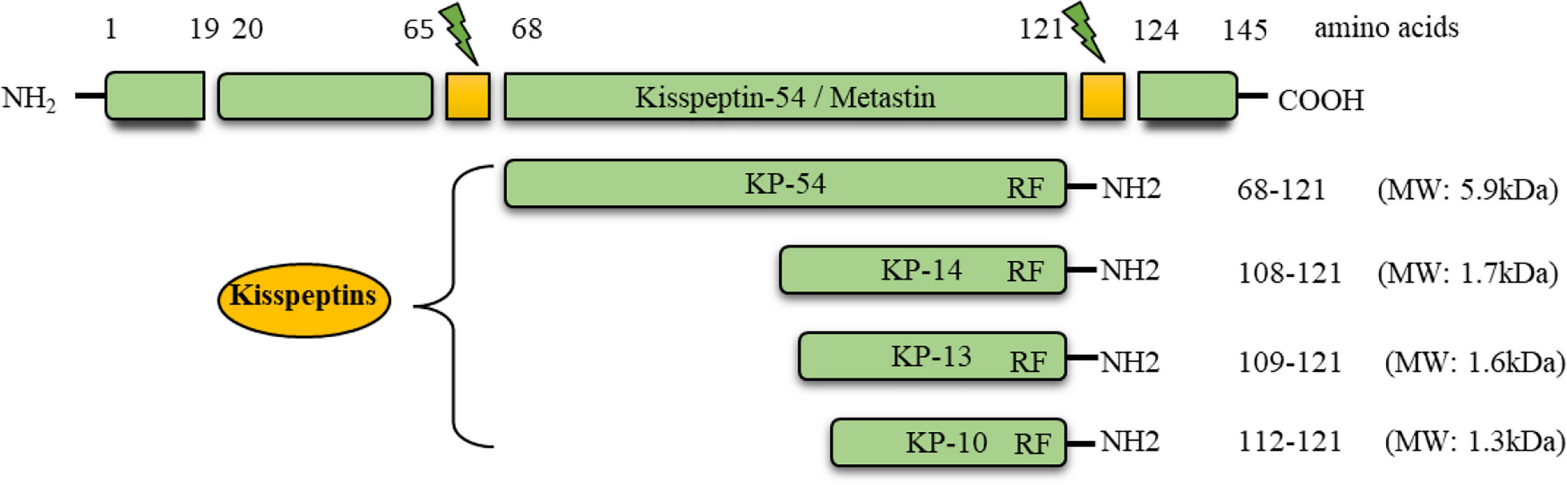
Conversion of prepro-kisspeptin into the active peptides. The prepro-kisspeptin is encoded by the *KISS1* gene in primates and encodes a 145 amino acid prepro hormone (MW: ~15.4 kDa). This polypeptide is cleaved into 54, 14, 13, and 10 amino acids. The C-terminal, 10 amino acid sequence is conserved in all the forms, and can activate the G-protein coupled receptor, GPR54 (KPR).

Kisspeptins (KPs) are a group of neuropeptides encoded by the *Kiss1* gene (1–3). The preprotein of 145 amino acids is cleaved into peptides containing 54 (52 in rodents), 14, 13, and 10 amino acids (2) (**Figure 1**). All of these peptides share the carboxy terminal 10 amino acids (RF amide in primates and RG amide in rodents), which bind to a G-protein coupled receptor (GPR54, encoded by the *Kiss1r* gene, referred as KPR in this article) with similar affinity (2, 3). Mouse and rat KPRs are nearly 95% identical, and approximately 85% identical to human KPRs (4).

KP was initially identified in a human melanoma cell line, and was suggested to be a metastasis suppressor (1). It was named metastatin, due to its potential antimetastatic and antiangiogenic properties (5). Subsequent studies demonstrated that the primary role of KPs lies within the hypothalamic-pituitary-gonadal (HPG) axis for reproduction (6–8). Individuals carrying inactivating mutations in either the KP or KPR genes suffer from hypogonadotropic hypogonadism (6–8). On the other hand, activating mutations of the KPR resulted in precocious puberty (9). These findings suggest that KP signaling system is crucial for a normal functioning HPG axis. The roles of KPs and KPRs in the HPG axis or reproduction have also been examined in experimental mouse and rat models by knocking out the *Kp* or *Kpr* gene (7, 10). Loss of KPs or KPRs disrupted GPN secretion, which affected gonadal development, steroidogenesis, as well as gametogenesis in both sexes (6–8, 10). Remarkably, *Kpr* knockout (*Kpr^KO^*) mouse models exhibited a more severe hypogonadism phenotype than the knockout of the *Kp* gene (*Kp^KO^*) (7, 10).

Expression of KPs in hypothalamic KP neurons is regulated by gonadal steroid hormones (11). However, during the pre-pubertal period, expression of KPs occurs independent of gonadal steroids, which switches to gonadal steroid dependent at puberty (11). KPs act on the KPRs in gonadotropin (GPN) releasing hormone (GnRH) neurons to induce the expression of GnRH (11). GnRH induces pituitary gonadotrophs to release the GPNs, follicle stimulating hormone (FSH) and luteinizing hormone (LH) (12–14). GPNs act on the gonads to promote specific hormonal and reproductive functions (6, 7, 15, 16). Thus, hypothalamic expression of KPs is essential for the gonadal development, onset of puberty, and maintenance of gonadal functions (6, 7, 12, 13, 15, 16).

Expression of KPs and KPRs has also been detected in several extrahypothalamic sites inside the brain as well as organs outside the brain (17–26). There have been various types of suggested functions for extrahypothalamic KPs in different organ systems (27–32). However, until recently, proven functions of extrahypothalamic KP-signaling has remained limited to pancreas, liver, and the ovary (33–36). KPs and KPRs are expressed in ovaries across species (27–32). KPs are expressed predominantly in rat granulosa cells (GCs), whereas KPRs are expressed mainly in rat oocytes (24). Intrafollicular KP levels rise with the stages of oocyte maturation and correlate with follicular estradiol concentration (37). KP-signaling has been found to be crucial for mouse oocyte survival (35, 36) as well as oocyte maturation and ovulation (35, 38). In women undergoing *in vitro* fertilization (IVF), administration of exogenous KPs or a KPR agonist have also been found to improve oocyte maturation (39–42). Additionally, several studies have demonstrated that treatment with KPs can increase *in vitro* maturation (IVM) of oocytes from different species (38, 43, 44). The purpose of the current review is to understand the role of hypothalamic and extrahypothalamic KPs in maturation of oocytes, *in vivo* and *in vitro*.

## Hypothalamic kisspeptins

KP-signaling represents a regulatory link between the gonadal steroid hormones and the GPNs. GPNs are secreted from the anterior pituitary gland in response to GnRH, and act on the gonads (45). In response to GPNs, the gonads produce steroid hormones including estradiol, progesterone, and testosterone (46). The gonadal steroid hormones in turn regulate the secretion of GPNs from the HP axis (46). GnRH neurons in the hypothalamus that produce GnRH to regulate GPN secretion from the anterior pituitary lack receptors for gonadal steroid hormones (47). Instead, the GnRH neurons express KPRs, whereas the KP neurons in the hypothalamus possess receptors for gonadal steroid hormones (46, 47). Gonadal steroids act on KP neurons to regulate the expression of KPs, and KPs act on the KPRs expressed in GnRH neurons to induce GnRH production (46, 47). Although there have been many suggested functions for hypothalamic KPs, the primary role of hypothalamic KPs is to regulate the GnRH secretion from the GnRH neurons.

### Hypothalamic kisspeptin mediated regulation of ovarian function

**Figure 2:**
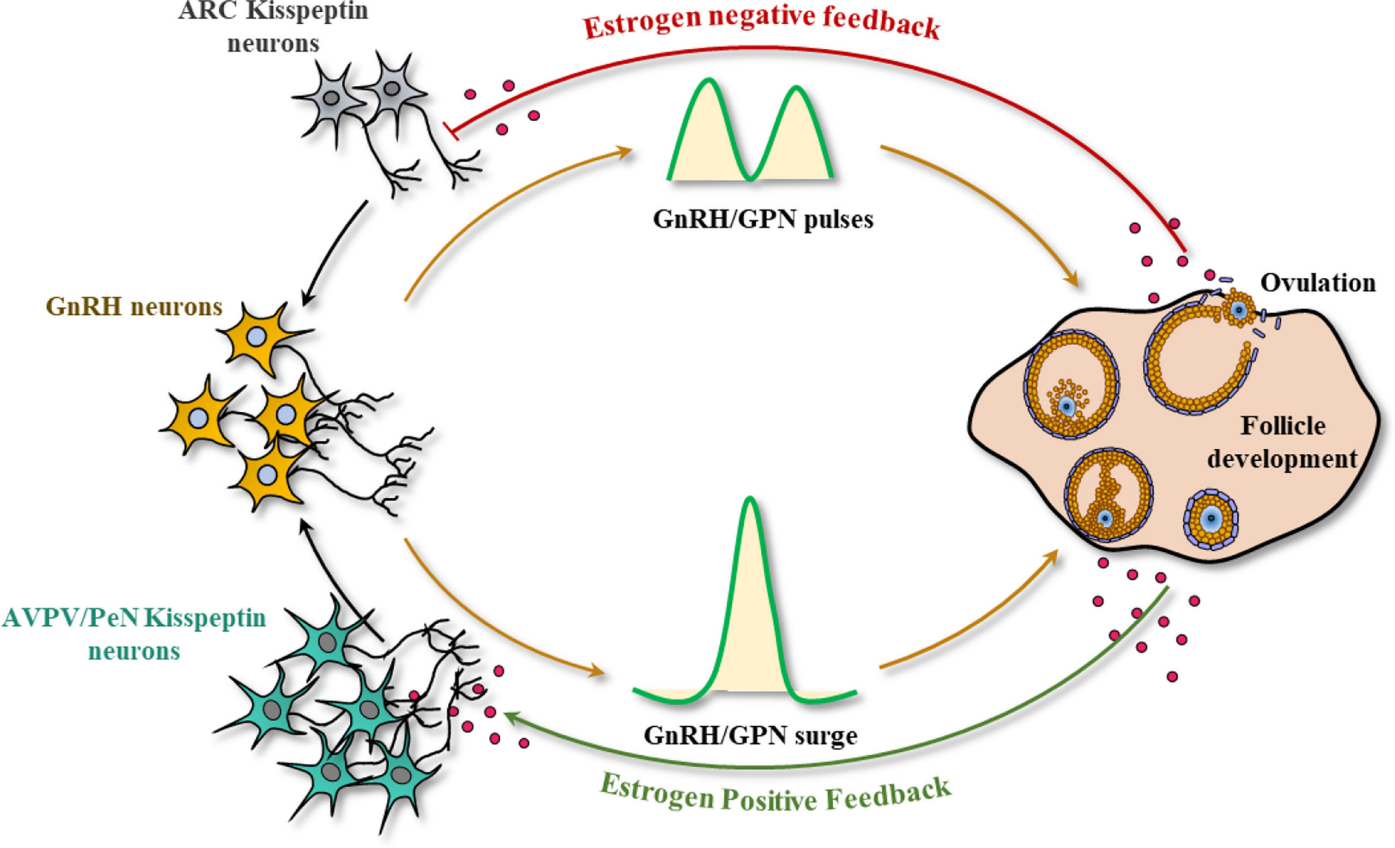
Kisspeptin signaling in the hypothalamic-pituitary-gonadal axis. In most mammals, there are two major sites of kisspeptin (KP) expression in the hypothalamus: the infundibular nucleus, and the nuclei in preoptic area (PeN) (arcuate nucleus (ARC) and anteroventral periventricular nucleus (AVPV) in rodents). Small amounts of estrogen synthesized from the ovary results in the activation of KP neurons in the infundibular/ARC nucleus, modulating the pulsatile secretion of GnRH and gonadotropins (GPNs). In contrast, an increased level of estrogen during the follicular phase (analogous to proestrus stage) activates the KP neurons in PeN/AVPV and inhibits the infundibular/ARC neurons, generating the GnRH/GPN surge, enabling oocyte maturation, and ovulation.

As aforementioned, hypothalamic KP-signaling is the key mediator of estrogen-induced GnRH and GPN secretion that regulates gonadal functions (48, 49). There are two major populations of hypothalamic KP neurons: one localized in the infundibular nucleus in primates (50, 51) and the other in the nuclei in the preoptic area (PeN) in ruminants (52–59) and primates (51, 60–63). The corresponding nuclei in rodents are located in arcuate [ARC] nucleus and the anteroventral periventricular [AVPV] nucleus respectively (64–68) (**Figure 2**).

While gonadal steroids negatively regulate KP expression from the infundibular/ARC nucleus, they positively regulate the KP neurons in the PeN/AVPV (69, 70). Estrogen receptor α (ERα) is the predominant estrogen receptor that regulates both positive and negative regulation of KP expression in hypothalamic KP neurons (64). The KP/Neurokinin B/dynorphin (KNDY) neurons serve as the GPN pulse generator (71). It was found that rescuing 20% of the KNDY neurons in the infundibular/ARC nucleus could recover the GPN pulse release in *Kp^KO^* rats (71). Pulsatile secretion of KPs from the infundibular/ARC nucleus results in pulsatile secretion of GnRH in both sexes. In contrast, bolus secretion of KPs from the PeN/AVPV nucleus in response to elevated estrogen level in females leads to preovulatory GnRH and subsequent GPN surge. While pulsatile secretion of GPNs from infundibular/ARC is important for ovarian follicle development, the preovulatory GPN surge from the PeN/AVPV nucleus is essential for the final stages of oocyte maturation and ovulation (69, 70).

### Sexual dimorphism in hypothalamic kisspeptin

Both the quantity and transcriptional activity of hypothalamic KP neurons are sexually dimorphic. Studies in human autopsy samples found that females have significantly higher numbers of KP synthesizing fibers in the hypothalamus compared to males (72). In addition, studies in mouse models found a sex difference in KP expression starting from fetal life, females always showing significantly higher levels (73). The sexually dimorphic feature is more prominent in the PeN/AVPV nucleus compared to the infundibular/ARC nucleus. A10-fold, female-dominance in the numbers of KP neurons is found in the PeN/AVPV (65). This prominent PeN/AVPV KP system in females is important for generating female-specific preovulatory GnRH and GPN surge (65, 74). An increased level of estrogens increases KP expression in PeN/AVPV nuclei and results in a preovulatory GPN surge, which is essential for oocyte maturation, ovulation, and formation of the corpus luteum (75).

### Hypothalamic kisspeptins and oocyte maturation

Although oocytes express KPRs, a widely accepted role of KPs in oocyte maturation is indirect, through induction of GnRH and GPN response from HP axis. KPs induce pulsatile GPN secretion from the infundibular/ARC nucleus, which results in FSH-induced ovarian follicle development to the antral state (69). Further development of the antral follicles to preovulatory Graafian follicles, maturation of oocytes, and ovulation are mediated by the LH surge induced by large amount of KPs released from the PeN/AVPV nucleus (69). Recent studies emphasize the importance of KPRs in oocytes for induction of oocyte maturation (35, 76). But it remains unknown whether hypothalamic KPs reach the ovarian follicles and act on the KPRs in the oocytes. On the other hand, the preovulatory GPN surge results in intraovarian KP expression, which may also act on the KPRs expressed in oocytes. The role of GC-derived KPs in oocyte maturation, however, has not been proven.

## Extrahypothalamic kisspeptins

It is well-established that KPs and KPRs play a critical role in regulation of GPN secretion from HP axis (6, 7, 10). Recent studies demonstrated a more expansive role for KP-signaling in various extrahypothalamic organs both inside and outside the brain (12, 13, 29). Several studies have found that the functions of extrahypothalamic KPs are also vital for regulation of metabolism and reproduction (33–36).

### Kisspeptin signaling in the pituitary gonadotrophs

The role of KPRs and KP-signaling in hypothalamic GnRH neurons regulating GnRH secretion and reproductive function in both sexes is widely recognized (6, 10, 48, 49, 77, 78). KPRs are also detected in the pituitary gland, but their roles remain largely unclear (79, 80). It was shown that KPs can activate KPRs in a pituitary gonadotroph cell line LβT2 and induce protein kinase C-dependent expression of FSHβ and LHβ (80). In contrast, a recent study has demonstrated that pituitary-specific knockout of KPRs reduced the FSH level in male mice, but not the LH level or testicular function (81). Neither GPN levels in female mice nor the reproductive function in male or female mice was affected following the loss of KPRs in pituitary gland (81). Thus, the clinical importance of KPRs in the pituitary is still under investigation.

### Extrahypothalamic kisspeptins and kisspeptin receptors outside the brain

**Figure 3:**
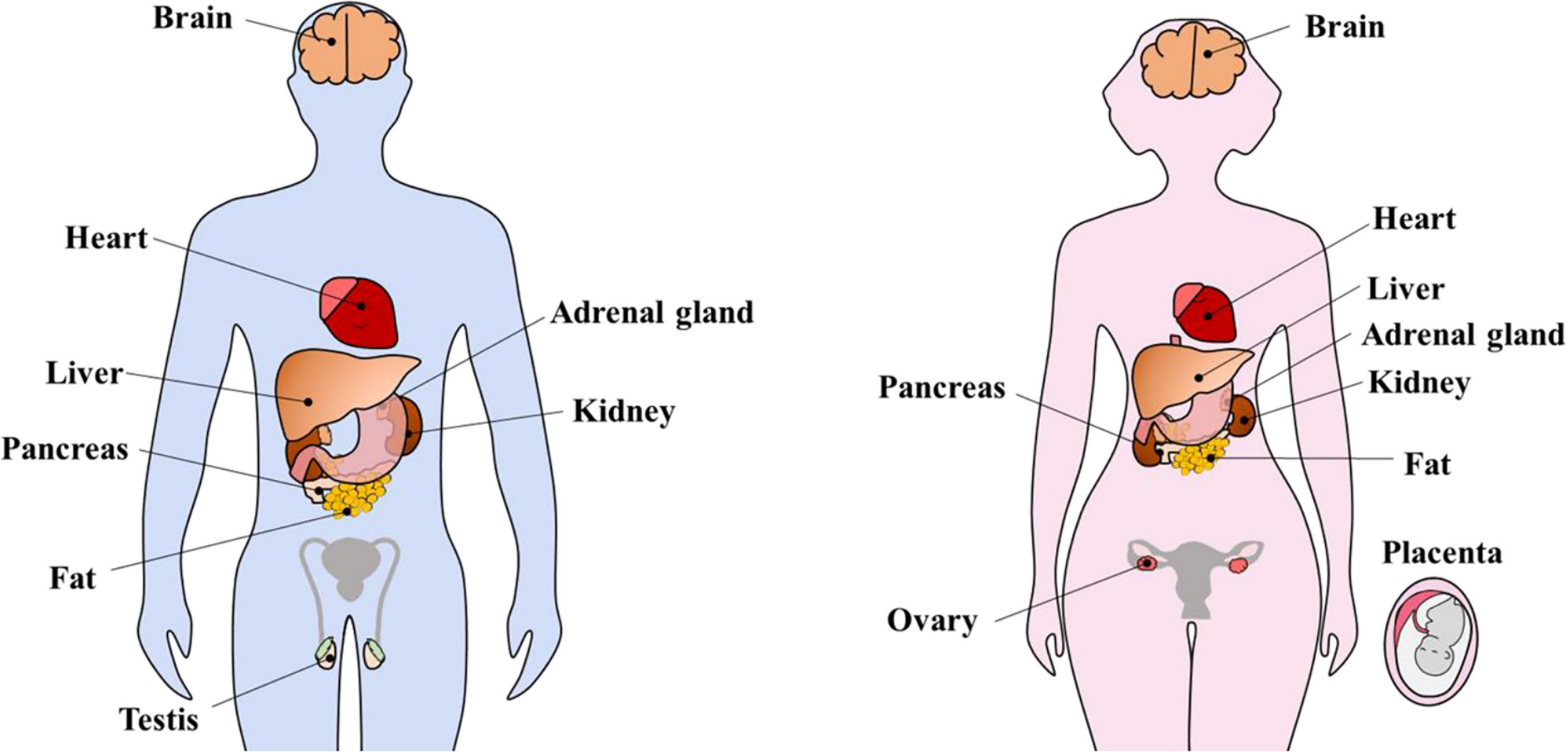
Extrahypothalamic kisspeptins and kisspeptin receptors. Kisspeptins (KPs) and kisspeptin receptors (KPRs) are expressed in several extrahypothalamic sites inside the brain as well as peripheral organs including heart, liver, adrenal gland, pancreas, kidney, fat, testis, and ovary. During pregnancy, KP expression increases tremendously, in part due to placental expression.

KP and KPRs have been detected in extrahypothalamic organs with a multitude of functions. It has been shown that KPs/KPRs are expressed in the liver (17, 82), pancreas (83, 84), adipose tissue (17–19), testis (17, 20, 21), ovary (22–24), adrenal gland (85), heart (86), uterus (87–89) and placenta (25, 31, 88) (**Figure 3**). Peripherally administered KP can act on hypothalamic GnRH neurons to induce GPN secretion (61, 90–92). Based on this finding, we can assume that KP produced in extrahypothalamic tissues may have a direct impact on hypothalamic GnRH neurons and GPN secretion, and *vice-versa*. Future studies will determine if hypothalamic or extrahypothalamic KPs play such a role.

### Role of extrahypothalamic kisspeptins inside and outside the brain

The major function of KP-signaling in the brain involves regulation of reproductive functions (48, 91). In addition to reproductive neuroendocrinology, KPs may serve diverse neurological functions inside the brain (93). While administration of exogenous KP enhanced memory (94), loss of KP-signaling has been associated with a reduced anxiety-related behavior in mice (95). Estrogen driven KP-signaling regulates glutamate neurotransmission to the infundibular/ARC nucleus, which controls feeding behavior (96, 97). In addition, KP-signaling regulates the pathophysiology in several extrahypothalamic organs outside the brain including liver, pancreas, and heart (84, 97–100). It regulates insulin secretion from the pancreas and maintains glucose homeostasis (33), lipid metabolism in the liver and control of non-alcoholic fatty changes (34), as well as oocyte maturation and ovulation in the ovaries important for female fertility (35, 36). KP-signaling has been suggested to play a role in spermatogenesis and activation of sperm (101). KPs can modulate intracellular Ca^2+^ influx, sperm motility, sperm hyperactivation, and the acrosome reaction in the human spermatozoa, that is critical for fertilization and IVF outcomes (32, 102). IVF rates were decreased after the treatment of sperm with KP antagonist KP234, which suggest the importance of KP-signaling in fertilization process (102). Further studies are required to determine if treatment of sperm with KPs can increase IVF. KPs and KPRs may also be involved in the regulation of cancer metastasis, vascular dynamics, implantation of embryos, and placental physiology, none of which are not yet understood clearly (27–32). Despite all these suggested functions, the only understood ones are limited to insulin secretion (100), metabolism, and ovarian folliculogenesis (33, 35).

### Extrahypothalamic kisspeptins and oocyte maturation

The ovary is one of the extrahypothalamic sites of KP and KPR expression (27–32). The *Kp* gene is expressed predominantly in rat GCs, whereas the *Kpr* gene is found predominantly in rat oocytes (24, 38). *Kp* mRNAs are expressed within the ovaries in response to the preovulatory GPN surge (22, 24, 103), and KP concentration increases in follicular fluid during oocyte maturation (37). Taken together, it was hypothesized that intrafollicular KPs can induce KP-signaling in oocytes and promote *in vivo* oocyte maturation (24, 38, 104). Very recently, an elegant study from the Tena-Sempere lab has presented evidence that KP-signaling in mouse oocytes is essential for follicle development and survival of mature ovarian follicles (35). The research group has also demonstrated that loss of KP-signaling in oocytes leads to premature ovarian insufficiency (**POI**) in oocyte-specific *Kpr^KO^* mice (35). Thus, the role of KPs in regulating female fertility can involve organ systems well beyond the HP axis.

## Kisspeptin signaling in the ovary

KPs are known inducers of GPNs in the HP axis (48). But within the ovary, GPNs act as inducers of KP expression from the GCs (24, 38). While the hypothalamic *Kp* gene is regulated by ERα (105), ovarian *Kp* expression is regulated by ERβ (24). GPNs act on the GPN receptors (FSHR and LHCGR), on follicular theca-interstitial cells (**TCs**) and GCs, which ultimately promote oocyte maturation (106). Ovarian KPs may also act on KPRs in oocytes to induce follicle development and oocyte maturation (35, 36). Nevertheless, both hypothalamic and ovarian KP signaling indirectly or directly act to induce oocyte maturation. The findings discussed in the following sections were obtained from rodent studies.

### Expression of kisspeptin and kisspeptin receptors

*Kp* and *Kpr* genes are expressed within the ovaries of diverse animal species including fish (107, 108), birds (109), rodents (22, 110, 111), canines (112, 113), cattle (114, 115), and primates (116). There has been some variation in reports regarding the relative expression of KPs or KPRs in oocytes and somatic cells (28). While some reports showed detection of KPs and KPRs in all ovarian cell types, others have reported that *Kp* mRNA is predominantly expressed in GCs, and KPRs are expressed in the oocytes (24, 38, 117). A marked upregulation in *Kp* gene expression was observed after the injection of human chorionic gonadotropin (hCG) into pregnant mare serum gonadotropin (PMSG) primed rats (24). The expression of the *Kp* gene in GCs was also dependent on the presence of ERβ (24). As expected, oocytes did not show the upregulation in *Kp* gene expression as they lack GPN receptors, and theca-interstitial cells (TCs) lacked *Kp* gene expression due to low levels of ERβ (24, 117–119). Indomethacin treatment that inhibits prostaglandin synthesis and prevents ovulation, also inhibited hCG-induced expression of the *Kp* gene, which suggests an essential role for cyclooxygenase and/or prostaglandins in *Kp* gene regulation (22, 116). Although *Kp* gene expression in hypothalamic KP neurons is independent of progesterone receptor (PGR), secretion of KPs from the KP neurons is regulated by PGR (120). Further studies are required to determine if PGR plays any role on intraovarian KP expression or secretion.

### Gonadotropin secretion and steroidogenesis

Expression of KPs in GCs and KPRs in oocytes suggest that KP-signaling may represent bidirectional signaling between the GCs and oocytes, for controlling follicle development, steroidogenesis, oocyte maturation, and ovulation (24). Hypothalamic KPs regulate the secretion of GPNs, which act on gonadal cells to regulate steroidogenesis. It has been shown that low serum KP levels is associated with a lower levels of serum estrogen and progesterone (121). A low level of KPs represses GnRH release from hypothalamus, decreases GPN secretion from pituitary gonadotrophs that ultimately affects the synthesis of progesterone and estrogen in follicular TCs and GCs (122). As previously stated, these findings indicate that KPs of extrahypothalamic origin, including ovarian-derived KPs, may have a significant effect on hypothalamic GnRH production and GPN secretion. In contrast, results with a mutant mouse model suggests that KP-signaling in the hypothalamic GnRH neurons is sufficient for the regulation of the hypothalamic pituitary ovarian (HPO) function (123). In contrast, a later study using the same mutant mouse model demonstrated that hypothalamic GnRH-specific expression of KPRs in global *Kpr^KO^* mice still suffer from abnormal ovarian ultrastructure and premature ovarian ageing (124). In another study, *Erβ^KO^* mouse ovaries were found to synthesize low levels of estrogens and the preovulatory GPN peak was remarkably diminished (125). Neither administration of exogenous estrogen could induce a natural GPN surge nor exogenous GPNs could mediate follicle maturation and ovulation in *Erβ^KO^* mice, but exchange transplantation of wildtype ovaries could resolve those defects (125). These findings suggest that wildtype ovaries express a crucial factor that is absent in the *Erβ^KO^* ovaries. Later, we identified that the ERβ-regulated crucial factor could be GC-derived KPs, which is absent in *Erβ^KO^* GCs (24, 38, 104).

### Follicle activation and follicle development

KP-signaling was found to be involved in both the initial and the cyclical recruitment of rat ovarian follicles (126). Administration of a KP antagonist, KP234, into rat ovaries increased primordial follicle activation (PFA) (126). Although the mechanism of increased PFA remains unclear, it was associated with decreased FSHR on preantral follicles that reduced the cyclic activation (126). However, administration of the KP antagonist decreased the numbers of mature follicles and ovulation, suggesting that KP-signaling plays a positive role in preovulatory follicle maturation (126). Ovarian expression of KPs increases during the preovulatory period (22), which correlates with the potential role KPs in preovulatory oocyte maturation. Administration of KPs in rats increases follicle maturation induced by hCG (116). In addition, *Kpr^KO^* mice were observed to have arrested follicle development even in the presence of GPNs (36). In *Erβ^K^* ^O^ rat ovaries, follicle development failed to progress beyond the antral stage, which is also associated with an absence of GPN-induced ovarian KP expression (127). It has been shown that KPs increase the number of type III follicles that originate from the preovulatory follicles and possess a large antral space (128–130). However, this response may in part be mediated by an indirect effect of KPs on the HP axis that induces GPNs.

### Oocyte maturation

**Figure 4:**
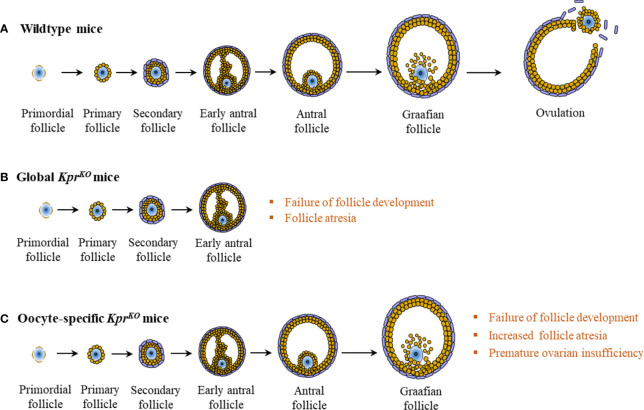
Intraovarian kisspeptin signaling. In wildtype mice, normal development of the ovarian follicle leads to ovulation **(A)**. However, in global *Kpr^KO^* mice, follicles fail to develop beyond the secondary or the early antral stage **(B)**. In contrast to wildtype or global *Kpr^KO^* mice, oocyte specific *Kpr^KO^* mouse ovaries display markedly increased follicular atresia at late stages of follicle development, resulting in progressive failure of ovulation and premature ovarian insufficiency at 4 to 6 months of age **(C)**.

While KPRs are expressed in oocytes, surrounding GCs express KPs (24). A recent mutant mouse study has demonstrated an essential role for KPRs in oocytes for oocyte maturation and ovulation (**Figure 4**) (35). A low level of KP is expressed in the ovary in the basal condition, which is upregulated during the preovulatory period in late proestrus of rats (22). Basal levels of KPs in GCs is upregulated by PMSG-induced activation of FSHR, which is further upregulated by hCG-induced activation of LHCGR (104, 127). In human ovaries, intrafollicular KP levels were found to be increased with the progress in follicle development and maturation (37, 131).

These expression patterns of KPs and KPRs suggest that KPs produced by the GCs act on the KPRs expressed in oocytes to mediate a physiological response during the preovulatory oocyte maturation (24). It is likely mediated by KP-activated protein kinases like ERK1/2 and selective mRNA degradation to increase the expression of necessary proteins (38).

### Ovulation

Over the past two decades, KPs have been recognized as the most potent activator of GnRH and GPN secretion in mice (49), rats (14, 132), sheep (48), goats (133), cattle (134), and humans (135). Hypothalamic KPs are essential for the preovulatory GPN surge, which is required for follicle maturation and ovulation. The autocrine and paracrine functions of KPs were further observed in an experiment in which oocyte-specific *Kpr^KO^* mice failed to ovulate (35, 136). Although the expression patterns of ovarian KPs and KPRs suggest a potential role in preovulatory follicle maturation and ovulation, this has not yet been determined experimentally. During ovarian aging, fewer follicles are recruited for activation and maturation, decreasing the number of healthy antral follicles available for ovulation (137, 138). Despite a reduced number of follicles, the ratio of corpora lutea to antral follicles increases with advanced age, indicating that ovulation is more efficient (137, 138). This is associated with an increased level of KPs in aging females, which might be due to reduced inhibition of infundibular/ARC nuclei due to a lower level of estrogens (137, 138). An elevated level of KPs leads to increased GPN secretion that improves follicle maturation and ovulation. Administration of the KP antagonist, KP234 not only affects follicle development, but also disrupts oocyte maturation and ovulation (138). Loss of KPRs in mouse oocytes also disrupted oocyte maturation and ovulation (35, 36). Moreover, several recent studies have demonstrated that KP stimulation can induce IVM of oocytes (38, 43), which supports a potential *in vivo* role of KP in oocyte maturation. However, further studies are necessary to prove the physiological role of direct intraovarian KP-signaling in oocyte maturation.

## Kisspeptin and *in vitro* fertilization of oocytes

**Figure 5:**
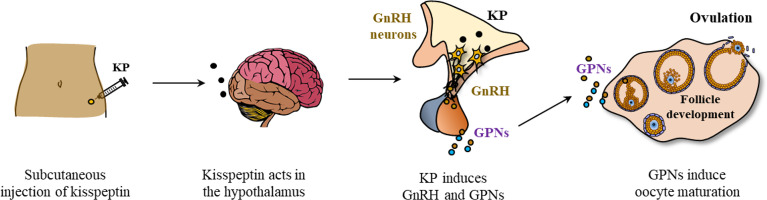
Kisspeptin induced oocyte maturation in patients undergoing *in vitro* fertilization. Exogenous kisspeptin (i.e., KP-54) administered at peripheral sites (subcutaneously) can reach the GnRH neurons in the hypothalamus and stimulate GnRH expression. GnRH acts on the pituitary gonadotrophs to induce gonadotropins (GPNs, LH and FSH) that act on the ovarian follicles to induce oocyte maturation and ovulation.

Based on the positive results in animal experiments following peripheral administration of KPs, KP-54 (54 amino acid form of human KP) was tested for its role in IVF. In several studies, administration of exogenous KP-54 was found to be a good option for inducing oocyte maturation (39, 40, 139, 140). A single dose of KP-54 injection could trigger oocyte maturation in women undergoing IVF (39) (**Figure 5**).

Circulating LH, FSH, and progesterone levels were elevated following the administration of exogenous KP-54 to women undergoing IVF (39). There was a dose-dependent increase in mature oocyte yield with kisspeptin dose, although both 6.4 and 12.8 nmol/kg are likely to represent doses near the top of the dose-response curve as LH rises were similar (39). In an *in vitro* study, treatment of granulosa lutein cells with KP-54 significantly increased the expression of GPN receptors (141). However, it needs to be determined whether KP-54 can also upregulate the expression of GPN receptors on GCs *in vivo*.

hCG is administered to achieve the final stages of oocyte maturation for IVF, but this carries some adverse effects. hCG can cause severe complications like ovarian hyperstimulation syndrome (OHSS) due to its prolonged half-life and potent stimulation of LHCGR. hCG induced excessive VEGF production in the ovaries plays a crucial role in the pathophysiology of OHSS (142, 143). KPs induce endogenous GnRH and GPNs, which may not stimulate ovarian production of excessive VEGF (40, 143). Moreover, KPs have been shown to inhibit ovarian VEGF expression (143, 144), that may alleviate the side effects of hCG administration. A recent study has found that a second injection of KP-54 could improve oocyte maturation in women at high risk of OHSS (139). This finding suggests that KP-54 might be considered a safe choice for women undergoing ARTs, especially those who have a higher risk for developing OHSS. However, further studies are required to compare the relative risks of hCG induced OHSS with that of the GnRH agonist and KP-54.

More recently, a KPR agonist oligopeptide, MVT-602, has been administered to healthy women as well as women with polycystic ovary syndrome (PCOS) or hypothalamic amenorrhea (HA) and compared the effects with that of KP-54 (42). In healthy women, MVT-602 induced LH secretion was similar to that of KP-54, but the effect exhibited a prolonged duration (42). While PCOS patients responded similarly to MVT-602 and KP-54, HA patients had an early response to MVT-602. These findings indicate that MVT-602 possesses a considerable therapeutic potential comparable to KP-54 (42).

## Kisspeptins and *in vitro* maturation of oocytes

IVM of oocytes is an advanced laboratory technique used for ARTs, where oocytes are collected before complete maturation. The partially mature oocytes are cultured *ex vivo* for further maturation. Once the *ex vivo* maturation is completed, the oocytes are fertilized *in vitro* and developing embryos are transferred to the uteri of the recipients. IVM is considered for younger females who have a larger follicle reserve. It requires minimal hormone administration, which reduces side effects including OHSS. IVM is recommended for patients with PCOS, as they are more susceptible to OHSS with IVF treatments. It is also very useful for women recovering from cancer, as cancer cells may get stimulated by the exposure to the hormones used in IVF.

**Figure 6:**
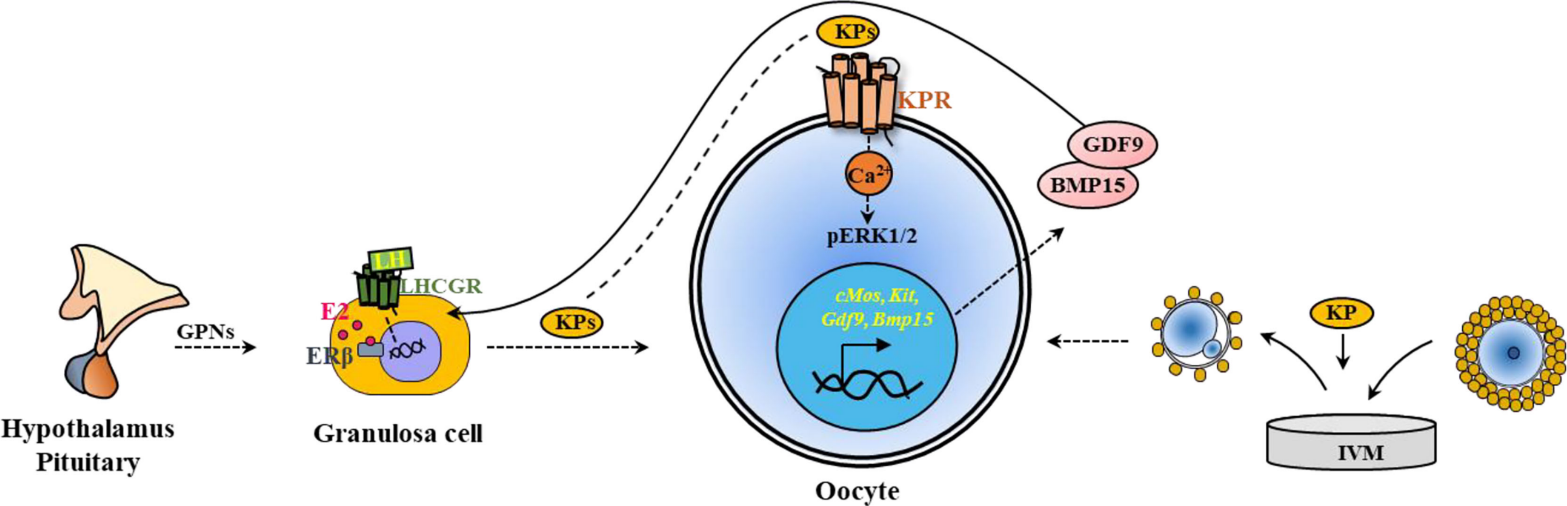
Kisspeptin induced *in vivo* and *in vitro* maturation of oocytes. Kisspeptins (KPs) mediate the maturation of ovarian follicles acting through hypothalamic-pituitary induction of gonadotropins (GPNs). GPNs act on granulosa cells and induce KP expression in the ovary. KPs can also act on the KPRs in the oocytes to induce oocyte maturation. Based on this strategy, KPs are included in the *in vitro* maturation (IVM) media to induce Ca^2+^ release and activate ERK1/2 in the oocytes. During IVM, KPs also upregulate the expression of oocyte genes like *cMos, Kit, Gdf9* and *Bmp15*, which improve oocyte maturation.

Both hypothalamic and intraovarian KPs have the potential to target maturation of oocytes, as they express KPRs. Hypothalamic KPs are known to act through GPN secretion, ovarian KPs can act directly on the oocytes and activate KPRs (**Figure 6**).

On the other hand, the oocytes express KPRs, and KPs have been tested for a role in IVM of oocytes. Several studies have demonstrated that supplementation of IVM media with KPs increase the rate of maturation of rat (38), sheep (43), and sow (44) oocytes (**Figure 6**).

KPRs are expressed in the oocytes and respond to KPs resulting in oocyte maturation (24, 38). Activation of KPRs augment intracellular Ca^2+^ release and activate mitogen-activated protein (MAP) kinases, extracellular signal-regulated kinase 1 and 2 (ERK1/2) in rat oocytes (38), similar to that was observed in hypothalamic neurons and luteal cells (145, 146). However, *in vitro* treatment of rat oocytes did not activate AKT (protein kinase B), which is also important for ovarian follicle activation (38). Studies have shown that inclusion of FSH in the oocyte culture media increase the expression of KPRs and augment KP-induced IVM of oocytes (44). IVM of both rat and sow oocytes was associated with upregulation of oocyte genes crucial for differentiation of GCs, and maturation of oocytes (38, 44). KP-10 treatment of rat and sow oocytes upregulated the relative expression of *cMos, Kit, Gdf9* and *Bmp15*, which are important for oocyte maturation (38, 44). As gene transcription is minimal during oocyte maturation, such differential expression of genes is likely due to selective degradation of mRNAs other than *cMos, Kit, Gdf9* and *Bmp15* (38, 44) (**Figure 6**).

KP-10 has been tested for IVM of rat, sheep, and sow oocytes (38, 43, 44). However, neither KP-10 nor KP-54 has been tested for IVM of human oocytes. In a recent study, the effects KPR agonist MVT-602 have been tested in cell lines and brain slices (42). MVT-602 induced inositol monophosphate (IP1) and Ca^2+^ signaling was comparable to that of human KP-54, however, the action potential firing of GnRH neurons in brain slices was longer than that of KP-54 (42). These findings suggest that both KP-54 as well as MVT-602 have a considerable potential for use in IVM of human oocytes.

## Ovarian diseases linked to kisspeptins

**Figure 7:**
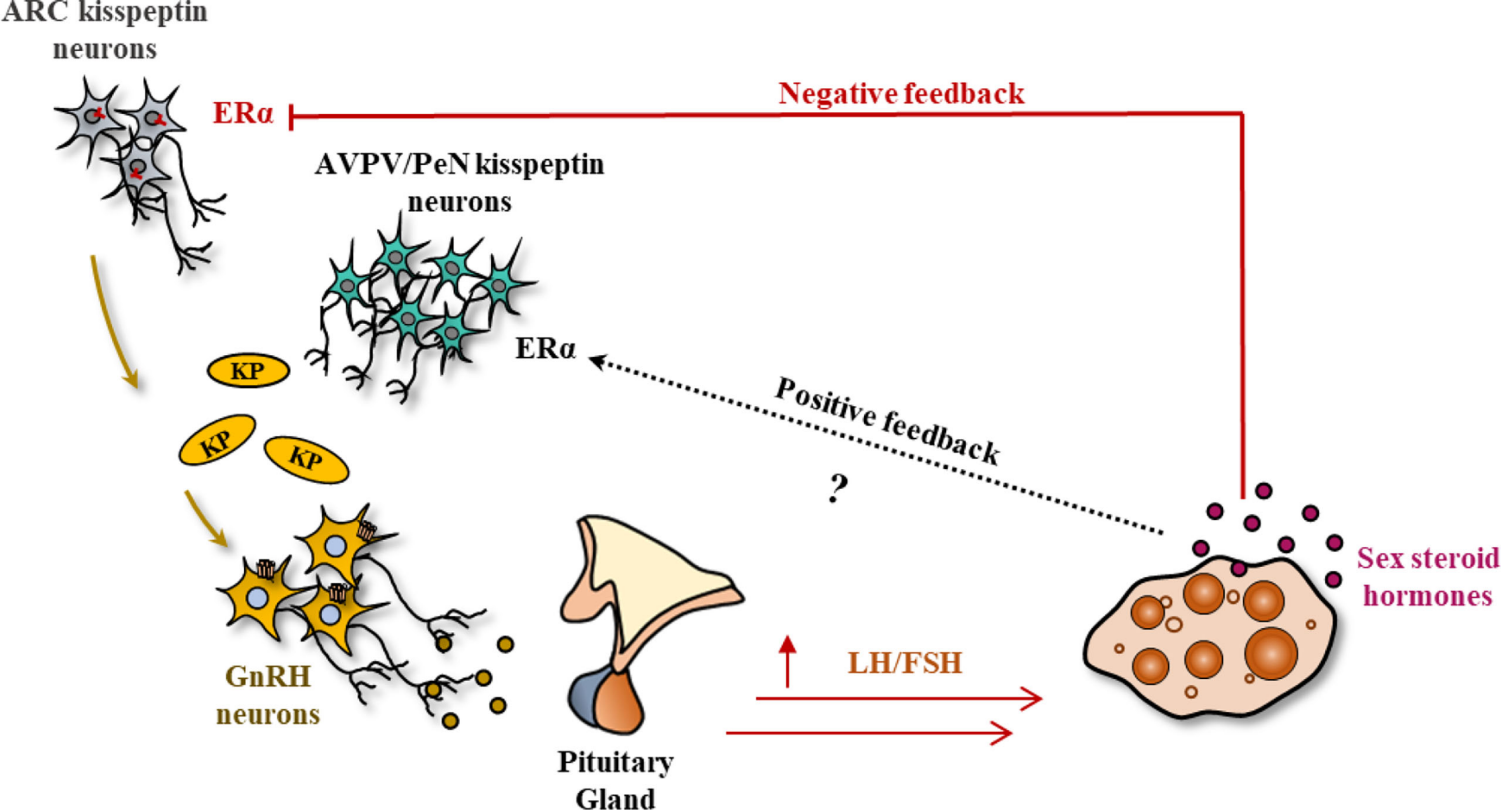
Polycystic ovary syndrome. Polycystic ovary syndrome (PCOS) is caused by increased levels of gonadotropins (GPNs, LH/FSH) due to the overstimulation by hypothalamic kisspeptins (KPs). Although the KP-response from the ARC nucleus is increased, it is suspected that PCOS lacks the estrogen induced KP surge from the PeN/AVPV nucleus required for generating the preovulatory GPN surge (unlike **Figure 2**). Lack of the preovulatory GPN surge in PCOS leads to a lack of follicle maturation and a failure of ovulation.

Dysregulation in KP signaling disrupts the HPO axis of neuroendocrine signaling, and negatively impacts ovarian function and fertility (28). Recent studies have linked several ovarian diseases to abnormal KP-signaling (7, 147, 148). PCOS, a common gonadal and metabolic disease, is associated with an elevated level of circulating KPs (7, 147–151). Some of the symptoms of PCOS include; endothelial dysfunction, elevated inflammatory markers, hormonal imbalances, and irregularities in the menstrual cycle (**Figure 7**) (152).

PCOS is commonly associated with high LH, but low FSH levels and decreased synthesis of estradiol as compared to their healthy counterparts (149, 150, 153). Exogenous KP treatment can also induce GPN response and rescue ovulation in a subset of PCOS patients (154). However, it is not yet determined whether any defects in intraovarian KP-signaling is linked to failure of follicle maturation in PCOS. Studies also suggest a potential link between PCOS and hypothyroidism, which is a common endocrine disorder (155, 156). It has been shown that maternal hypothyroidism can reduce the KPs and KPRs expression in placenta (157). Thus, it will be clinically important to know if thyroid hormones also regulate KP and KPR expressions within the ovary.

Development of precocious puberty has been linked to hypothalamic KP signaling (158). An aberrant gain of KP expression or KPR signaling may lead to precocious puberty (159). KPs have been proved to be a major therapeutic remedy in assisting women with reproductive and infertility issues (40–42, 139, 160). However, hypothalamic amenorrhea, which is associated with deficiency in GPN release and low KP levels, does not respond well with exogenous KP-54 administration. Despite an initial positive response, high dose KPs result in desensitization after a few weeks (161).

## Summary and conclusions

While the hypothalamic KPs regulate GPN secretion (6, 7, 10), GPNs activate KP secretion in the ovary (22, 24). During the last 25 years, KP/KPR studies have been focused primarily on the hypothalamic KP-signaling. The hypothalamic role of KP-signaling is well accepted but the role of ovarian KP/KPR remains largely unknown. Recent studies have emphasized the importance of KP-signaling in several extrahypothalamic sites (35, 38). However, the majority of extrahypothalamic functions remain unclear. Administration of KP-54 (54 amino acid human KPs) to women undergoing *in vitro* fertilization was found to trigger maturation of oocytes (39). Patients at high risk of OHSS have been successfully treated with KP-54 that can replace hCG (40). A recent study has also evaluated a KPR-agonist, MVT-602, which showed promising results similar to that of KP-54 (41, 42).

In most instances, oocyte maturation effects of exogenous KPs or the KPR-agonist have been attributed to the induction of hypothalamic GnRH and pituitary GPNs (39, 40, 42). However, KPs and KPRs are also expressed in the ovaries, which have been shown to play an essential role oocyte maturation and ovulation (24, 35, 38). Nevertheless, it remains undetermined if exogenous KPs or KPs of hypothalamic or extraovarian origins can act on the KPRs in oocytes. Oocytes express KPRs and supplementation of IVM media with KPs was found to increase the rate of IVM (38, 43, 44). KP stimulation during IVM increased Ca^2+^ release, MAP kinase (ERK1/2) activation, and upregulation of oocyte genes that promoted oocyte maturation (38, 43, 44). Moreover, FSH has been demonstrated to upregulate the expression of KPRs and augment KP-induced IVM of oocytes, which offers an opportunity to include both FSH and KPs in IVM media (44).

Despite the promising results of KP-induced *in vivo* and *in vitro* oocyte maturation, the growth of blastocysts and trophoblast outgrowth was reduced by incubation with kisspeptin in an *in vitro* study (44). Studies employing intraovarian kisspeptin administration or antagonism, reveal that kisspeptin has a role in reducing acquisition of FSH receptors and increased corpora lutea, signifying an important role in follicular development and ovulation during reproductive aging  (138). Most importantly, further studies are required to evaluate whether KP stimulated mature oocytes maintain their normal fertilization competence and developmental potential. We emphasize that elucidating the direct *in vivo* or *in vitro* roles of KPs or KP agonists in oocyte maturation will help develop novel KP-based strategies to improve the ARTs.

## Author contributions

This review article and illustrations were prepared by SM, EL, ID, and SU. M.A.R planned structure of the article and edited the content. VC and PF reviewed the manuscript and made valuable corrections. PF editing the revised manuscript and language correction. All authors approved the contents of the revised manuscript.

## Funding

This work was supported in part by the COBRE (P30 GM122731), K-INBRE (P20 GM103418), and NIH R21 HD105095 grant funding.

## Acknowledgments

The authors acknowledge Nathan Gaid, a Summer Trainee in Fields lab, for reading the revised manuscript and suggesting some grammatical correction.

## Conflict of interest

The authors declare that the research was conducted in the absence of any commercial or financial relationships that could be construed as a potential conflict of interest.

## Publisher’s note

All claims expressed in this article are solely those of the authors and do not necessarily represent those of their affiliated organizations, or those of the publisher, the editors and the reviewers. Any product that may be evaluated in this article, or claim that may be made by its manufacturer, is not guaranteed or endorsed by the publisher.

## References

[1] Lee JH Miele ME Hicks DJ Phillips KK Trent JM Weissman BE. KiSS-1, a novel human malignant melanoma metastasis-suppressor gene. J Natl Cancer Inst (1996) 88:1731–7. doi: 10.1093/jnci/88.23.1731 8944003

[2] Kotani M Detheux M Vandenbogaerde A Communi D Vanderwinden JM Le Poul E. The metastasis suppressor gene KiSS-1 encodes kisspeptins, the natural ligands of the orphan G protein-coupled receptor GPR54. J Biol Chem (2001) 276:34631–6. doi: 10.1074/jbc.M104847200 11457843

[3] Stafford LJ Xia C Ma W Cai Y Liu M. Identification and characterization of mouse metastasis-suppressor KiSS1 and its G-protein-coupled receptor. Cancer Res (2002) 62:5399–404. 12359743

[4] Clements MK McDonald TP Wang R Xie G O'Dowd BF George SR. FMRFamide-related neuropeptides are agonists of the orphan G-protein-coupled receptor GPR54. Biochem Biophys Res Commun (2001) 284:1189–93. doi: 10.1006/bbrc.2001.5098 11414709

[5] Liu N Lapcevich RK Underhill CB Han Z Gao F Swartz G. Metastatin: a hyaluronan-binding complex from cartilage that inhibits tumor growth. Cancer Res (2001) 61:1022–8. 11221828

[6] de Roux N Genin E Carel JC Matsuda F Chaussain JL Milgrom E. Hypogonadotropic hypogonadism due to loss of function of the KiSS1-derived peptide receptor GPR54. Proc Natl Acad Sci USA (2003) 100:10972–6. doi: 10.1073/pnas.1834399100 PMC196911 12944565

[7] Seminara SB Messager S Chatzidaki EE Thresher RR Acierno JS Jr. Shagoury JK. The GPR54 gene as a regulator of puberty. New Engl J Med (2003) 349:1614–27. doi: 10.1056/NEJMoa035322 14573733

[8] Topaloglu AK Tello JA Kotan LD Ozbek MN Yilmaz MB Erdogan S. Inactivating KISS1 mutation and hypogonadotropic hypogonadism. N Engl J Med (2012) 366:629–35. doi: 10.1056/NEJMoa1111184 22335740

[9] Teles MG Bianco SD Brito VN Trarbach EB Kuohung W Xu S. GPR54-activating mutation in a patient with central precocious puberty. N Engl J Med (2008) 358:709–15. doi: 10.1056/NEJMoa073443 PMC2859966 18272894

[10] Funes S Hedrick JA Vassileva G Markowitz L Abbondanzo S Golovko A. The KiSS-1 receptor GPR54 is essential for the development of the murine reproductive system. Biochem Biophys Res Commun (2003) 312:1357–63. doi: 10.1016/j.bbrc.2003.11.066 14652023

[11] Guerriero KA Keen KL Millar RP Terasawa E. Developmental changes in GnRH release in response to kisspeptin agonist and antagonist in female rhesus monkeys (Macaca mulatta): implication for the mechanism of puberty. Endocrinology (2012) 153:825–36. doi: 10.1210/en.2011-1565 PMC3275383 22166978

[12] Kauffman AS. Coming of age in the kisspeptin era: sex differences, development, and puberty. Mol Cell Endocrinol (2010) 324:51–63. doi: 10.1016/j.mce.2010.01.017 20083160 PMC2902563

[13] Oakley AE Clifton DK Steiner RA. Kisspeptin signaling in the brain. Endocrine Rev (2009) 30:713–43. doi: 10.1210/er.2009-0005 PMC2761114 19770291

[14] Irwig MS Fraley GS Smith JT Acohido BV Popa SM Cunningham MJ. Kisspeptin activation of gonadotropin releasing hormone neurons and regulation of KiSS-1 mRNA in the male rat. Neuroendocrinology (2004) 80:264–72. doi: 10.1159/000083140 15665556

[15] Kauffman AS Park JH McPhie-Lalmansingh AA Gottsch ML Bodo C Hohmann JG. The kisspeptin receptor GPR54 is required for sexual differentiation of the brain and behavior. J Neurosci (2007) 27:8826–35. doi: 10.1523/jneurosci.2099-07.2007 PMC6672184 17699664

[16] Lapatto R Pallais JC Zhang D Chan YM Mahan A Cerrato F. Kiss1-/- mice exhibit more variable hypogonadism than Gpr54-/- mice. Endocrinology (2007) 148:4927–36. doi: 10.1210/en.2007-0078 17595229

[17] Dudek M Kołodziejski PA Pruszyńska-Oszmałek E Sassek M Ziarniak K Nowak KW. Effects of high-fat diet-induced obesity and diabetes on Kiss1 and GPR54 expression in the hypothalamic-pituitary-gonadal (HPG) axis and peripheral organs (fat, pancreas and liver) in male rats. Neuropeptides (2016) 56:41–9. doi: 10.1016/j.npep.2016.01.005 26853724

[18] Brown RE Imran SA Ur E Wilkinson M. KiSS-1 mRNA in adipose tissue is regulated by sex hormones and food intake. Mol Cell Endocrinol (2008) 281:64–72. doi: 10.1016/j.mce.2007.10.011 18069123

[19] Cockwell H Wilkinson DA Bouzayen R Imran SA Brown R Wilkinson M. KISS1 expression in human female adipose tissue. Arch Gynecol Obstet (2013) 287:143–7. doi: 10.1007/s00404-012-2514-0 22899305

[20] Salehi S Adeshina I Chen H Zirkin BR Hussain MA Wondisford F. Developmental and endocrine regulation of kisspeptin expression in mouse leydig cells. Endocrinology (2015) 156:1514–22. doi: 10.1210/en.2014-1606 PMC4399318 25635620

[21] Irfan S Ehmcke J Shahab M Wistuba J Schlatt S. Immunocytochemical localization of kisspeptin and kisspeptin receptor in the primate testis. J Med Primatol (2016) 45:105–11. doi: 10.1111/jmp.12212 26987570

[22] Castellano JM Gaytan M Roa J Vigo E Navarro VM Bellido C. Expression of KiSS-1 in rat ovary: putative local regulator of ovulation? Endocrinology (2006) 147:4852–62. doi: 10.1210/en.2006-0117 16825322

[23] Yamasaki M Kuwahara A Iwasa T Yamamoto Y Taniguchi Y Yano Y. Development-related changes in the expression of the ovarian Kiss1 and Kiss1r genes and their sensitivity to human chorionic gonadotropin in prepubertal female rats. J Reprod Dev (2017) 63:409–14. doi: 10.1262/jrd.2016-179 PMC5593092 28552864

[24] Chakravarthi VP Khristi V Ghosh S Yerrathota S Dai E Roby KF. ESR2 is essential for gonadotropin-induced Kiss1 expression in granulosa cells. Endocrinology (2018) 159:3860–73. doi: 10.1210/en.2018-00608 PMC6260246 30277501

[25] Horikoshi Y Matsumoto H Takatsu Y Ohtaki T Kitada C Usuki S. Dramatic elevation of plasma metastin concentrations in human pregnancy: metastin as a novel placenta-derived hormone in humans. J Clin Endocrinol Metab (2003) 88:914–9. doi: 10.1210/jc.2002-021235 12574233

[26] Bowe JE Hill TG Hunt KF Smith LI Simpson SJ Amiel SA. A role for placental kisspeptin in beta cell adaptation to pregnancy. JCI Insight (2019) 4:e124540. doi: 10.1172/jci.insight.124540 PMC6824306 31619585

[27] Cejudo Roman A Pinto FM Dorta I Almeida TA Hernandez M Illanes M. Analysis of the expression of neurokinin b, kisspeptin, and their cognate receptors NK3R and KISS1R in the human female genital tract. Fertility sterility (2012) 97:1213–9. doi: 10.1016/j.fertnstert.2012.02.021 22424618

[28] Hu KL Zhao H Chang HM Yu Y Qiao J. Kisspeptin/Kisspeptin receptor system in the ovary. Front Endocrinol (2017) 8:365. doi: 10.3389/fendo.2017.00365 PMC5758547 29354093

[29] Dudek M Ziarniak K Sliwowska JH. Kisspeptin and metabolism: The brain and beyond. Front Endocrinol (2018) 9:145. doi: 10.3389/fendo.2018.00145 PMC5911457 29713310

[30] Hu KL Chang HM Zhao HC Yu Y Li R Qiao J. Potential roles for the kisspeptin/kisspeptin receptor system in implantation and placentation. Hum Reprod Update (2019) 25:326–43. doi: 10.1093/humupd/dmy046 PMC6450039 30649364

[31] Terao Y Kumano S Takatsu Y Hattori M Nishimura A Ohtaki T. Expression of KiSS-1, a metastasis suppressor gene, in trophoblast giant cells of the rat placenta. Biochim Biophys Acta (2004) 1678:102–10. doi: 10.1016/j.bbaexp.2004.02.005 15157736

[32] Pinto FM Cejudo-Roman A Ravina CG Fernandez-Sanchez M Martin-Lozano D Illanes M. Characterization of the kisspeptin system in human spermatozoa. Int J Andrology (2012) 35:63–73. doi: 10.1111/j.1365-2605.2011.01177.x 21651574

[33] Izzi-Engbeaya C Comninos AN Clarke SA Jomard A Yang L Jones S. The effects of kisspeptin on β-cell function, serum metabolites and appetite in humans. Diabetes Obes Metab (2018) 20:2800–10. doi: 10.1111/dom.13460 PMC6282711 29974637

[34] Guzman S Dragan M Kwon H de Oliveira V Rao S Bhatt V. Targeting hepatic kisspeptin receptor ameliorates non-alcoholic fatty liver disease in a mouse model. J Clin Invest (2022) 132:e145889. doi: 10.1172/jci145889 35349482 PMC9106350

[35] Ruohonen ST Gaytan F Usseglio Gaudi A Velasco I Kukoricza K Perdices-Lopez C. Selective loss of kisspeptin signaling in oocytes causes progressive premature ovulatory failure. Hum Reprod (Oxford England) (2022) 37:806–21. doi: 10.1093/humrep/deab287 PMC8971646 35037941

[36] Gaytan F Garcia-Galiano D Dorfman MD Manfredi-Lozano M Castellano JM Dissen GA. Kisspeptin receptor haplo-insufficiency causes premature ovarian failure despite preserved gonadotropin secretion. Endocrinology (2014) 155:3088–97. doi: 10.1210/en.2014-1110 PMC4611053 24885574

[37] Taniguchi Y Kuwahara A Tachibana A Yano Y Yano K Yamamoto Y. Intra-follicular kisspeptin levels are related to oocyte maturation and gonadal hormones in patients who are undergoing assisted reproductive technology. Reprod Med Biol (2017) 16:380–5. doi: 10.1002/rmb2.12056 PMC5715899 29259492

[38] Chakravarthi VP Ghosh S Housami SM Wang H Roby KF Wolfe MW. ERβ regulated ovarian kisspeptin plays an important role in oocyte maturation. Mol Cell Endocrinol (2021) 527:111208. doi: 10.1016/j.mce.2021.111208 33592287 PMC8906370

[39] Jayasena CN Abbara A Comninos AN Nijher GM Christopoulos G Narayanaswamy S. Kisspeptin-54 triggers egg maturation in women undergoing *in vitro* fertilization. J Clin Invest (2014) 124:3667–77. doi: 10.1172/jci75730 PMC4109525 25036713

[40] Abbara A Jayasena CN Christopoulos G Narayanaswamy S Izzi-Engbeaya C Nijher GM. Efficacy of kisspeptin-54 to trigger oocyte maturation in women at high risk of ovarian hyperstimulation syndrome (OHSS) during *In vitro* fertilization (IVF) therapy. J Clin Endocrinol Metab (2015) 100:3322–31. doi: 10.1210/jc.2015-2332 PMC4570165 26192876

[41] Abbara A Clarke S Chia Eng P Phylactou M Chia G Yang L. A single bolus of the kisspeptin analogue, MVT-602, induces a more prolonged LH surge than kisspeptin-54 during the follicular phase of healthy women. Fertil Steril (2018) 110:e103. doi: 10.1016/j.fertnstert.2018.07.310

[42] Abbara A Eng PC Phylactou M Clarke SA Richardson R Sykes CM. Kisspeptin receptor agonist has therapeutic potential for female reproductive disorders. J Clin Invest (2020) 130:6739–53. doi: 10.1172/jci139681 PMC7685751 33196464

[43] Byri P Gangineni A Reddy KR Raghavender KBP. Effect of kisspeptin on *in vitro* maturation of sheep oocytes. Veterinary World (2017) 10:276–80. doi: 10.14202/vetworld.2017.276-280 PMC5387653 28435188

[44] Saadeldin IM Koo OJ Kang JT Kwon DK Park SJ Kim SJ. Paradoxical effects of kisspeptin: it enhances oocyte *in vitro* maturation but has an adverse impact on hatched blastocysts during *in vitro* culture. Reproduction fertility Dev (2012) 24:656–68. doi: 10.1071/rd11118 22697116

[45] Sivalingam M Ogawa S Trudeau VL Parhar IS. Conserved functions of hypothalamic kisspeptin in vertebrates. Gen Comp Endocrinol (2022) 317:113973. doi: 10.1016/j.ygcen.2021.113973 34971635

[46] Eshkol A Lunenfeld B Insler V. The effect of sex steroids on pituitary responsiveness to gonadotropin releasing hormone. J Steroid Biochem (1975) 6:1061–6. doi: 10.1016/0022-4731(75)90350-7 1100906

[47] Shivers BD Harlan RE Morrell JI Pfaff DW. Absence of oestradiol concentration in cell nuclei of LHRH-immunoreactive neurones. Nature (1983) 304:345–7. doi: 10.1038/304345a0 6348552

[48] Messager S Chatzidaki EE Ma D Hendrick AG Zahn D Dixon J. Kisspeptin directly stimulates gonadotropin-releasing hormone release *via* G protein-coupled receptor 54. Proc Natl Acad Sci USA (2005) 102:1761–6. doi: 10.1073/pnas.0409330102 PMC545088 15665093

[49] Gottsch ML Cunningham MJ Smith JT Popa SM Acohido BV Crowley WF. A role for kisspeptins in the regulation of gonadotropin secretion in the mouse. Endocrinology (2004) 145:4073–7. doi: 10.1210/en.2004-0431 15217982

[50] Gottsch ML Popa SM Lawhorn JK Qiu J Tonsfeldt KJ Bosch MA. Molecular properties of Kiss1 neurons in the arcuate nucleus of the mouse. Endocrinology (2011) 152:4298–309. doi: 10.1210/en.2011-1521 PMC3199004 21933870

[51] Rometo A Krajewski S Voytko M Rance N. Hypertrophy and increased kisspeptin gene expression in the hypothalamic infundibular nucleus of postmenopausal women and ovarectomized monkeys. J Clin Endocrinol Metab (2007) 92:2744–50. doi: 10.1210/jc.2007-0553 17488799

[52] Goodman RL Lehman MN Smith JT Coolen LM de Oliveira CV Jafarzadehshirazi MR. Kisspeptin neurons in the arcuate nucleus of the ewe express both dynorphin a and neurokinin b. Endocrinology (2007) 148:5752–60. doi: 10.1210/en.2007-0961 17823266

[53] Hassaneen A Naniwa Y Suetomi Y Matsuyama S Kimura K Ieda N. Immunohistochemical characterization of the arcuate kisspeptin/neurokinin b/dynorphin (KNDy) and preoptic kisspeptin neuronal populations in the hypothalamus during the estrous cycle in heifers. J Reprod Dev (2016) 62:471–7. doi: 10.1262/jrd.2016-075 PMC5081734 27349533

[54] Ohkura S Takase K Matsuyama S Mogi K Ichimaru T Wakabayashi Y. Gonadotrophin-releasing hormone pulse generator activity in the hypothalamus of the goat. J Neuroendocrinol (2009) 21:813–21. doi: 10.1111/j.1365-2826.2009.01909.x 19678868

[55] Smith JT Li Q Pereira A Clarke IJ. Kisspeptin neurons in the ovine arcuate nucleus and preoptic area are involved in the preovulatory luteinizing hormone surge. Endocrinology (2009) 150:5530–8. doi: 10.1210/en.2009-0712 19819940

[56] Matsuda F Nakatsukasa K Suetomi Y Naniwa Y Ito D Inoue N. The luteinising hormone surge-generating system is functional in male goats as in females: involvement of kisspeptin neurones in the medial preoptic area. J Neuroendocrinol (2015) 27:57–65. doi: 10.1111/jne.12235 25367275

[57] Estrada KM Clay CM Pompolo S Smith JT Clarke IJ. Elevated KiSS-1 expression in the arcuate nucleus prior to the cyclic preovulatory gonadotrophin-releasing hormone/lutenising hormone surge in the ewe suggests a stimulatory role for kisspeptin in oestrogen-positive feedback. J Neuroendocrinol (2006) 18:806–9. doi: 10.1111/j.1365-2826.2006.01485.x 16965299

[58] Franceschini I Lomet D Cateau M Delsol G Tillet Y Caraty A. Kisspeptin immunoreactive cells of the ovine preoptic area and arcuate nucleus co-express estrogen receptor alpha. Neurosci Lett (2006) 401:225–30. doi: 10.1016/j.neulet.2006.03.039 16621281

[59] Smith JT Rao A Pereira A Caraty A Millar RP Clarke IJ. Kisspeptin is present in ovine hypophysial portal blood but does not increase during the preovulatory luteinizing hormone surge: evidence that gonadotropes are not direct targets of kisspeptin in vivo. Endocrinology (2008) 149:1951–9. doi: 10.1210/en.2007-1425 18162520

[60] Watanabe Y Uenoyama Y Suzuki J Takase K Suetomi Y Ohkura S. Oestrogen-induced activation of preoptic kisspeptin neurones may be involved in the luteinising hormone surge in male and female Japanese monkeys. J Neuroendocrinol (2014) 26:909–17. doi: 10.1111/jne.12227 25283748

[61] Shahab M Mastronardi C Seminara SB Crowley WF Ojeda SR Plant TM. Increased hypothalamic GPR54 signaling: a potential mechanism for initiation of puberty in primates. Proc Natl Acad Sci USA (2005) 102:2129–34. doi: 10.1073/pnas.0409822102 PMC548549 15684075

[62] Rometo AM Krajewski SJ Voytko ML Rance NE. Hypertrophy and increased kisspeptin gene expression in the hypothalamic infundibular nucleus of postmenopausal women and ovariectomized monkeys. J Clin Endocrinol Metab (2007) 92:2744–50. doi: 10.1210/jc.2007-0553 17488799

[63] Smith JT Shahab M Pereira A Pau KY Clarke IJ. Hypothalamic expression of KISS1 and gonadotropin inhibitory hormone genes during the menstrual cycle of a non-human primate. Biol Reprod (2010) 83:568–77. doi: 10.1095/biolreprod.110.085407 PMC2957156 20574054

[64] Smith JT Cunningham MJ Rissman EF Clifton DK Steiner RA. Regulation of Kiss1 gene expression in the brain of the female mouse. Endocrinology (2005) 146:3686–92. doi: 10.1210/en.2005-0488 15919741

[65] Clarkson J Herbison AE. Postnatal development of kisspeptin neurons in mouse hypothalamus; sexual dimorphism and projections to gonadotropin-releasing hormone neurons. Endocrinology (2006) 147:5817–25. doi: 10.1210/en.2006-0787 PMC6098691 16959837

[66] Smith JT Popa SM Clifton DK Hoffman GE Steiner RA. Kiss1 neurons in the forebrain as central processors for generating the preovulatory luteinizing hormone surge. J Neurosci (2006) 26:6687–94. doi: 10.1523/jneurosci.1618-06.2006 PMC6673844 16793876

[67] Adachi S Yamada S Takatsu Y Matsui H Kinoshita M Takase K. Involvement of anteroventral periventricular metastin/kisspeptin neurons in estrogen positive feedback action on luteinizing hormone release in female rats. J Reprod Dev (2007) 53:367–78. doi: 10.1262/jrd.18146 17213691

[68] Takase K Uenoyama Y Inoue N Matsui H Yamada S Shimizu M. Possible role of oestrogen in pubertal increase of Kiss1/kisspeptin expression in discrete hypothalamic areas of female rats. J Neuroendocrinol (2009) 21:527–37. doi: 10.1111/j.1365-2826.2009.01868.x 19500223

[69] Marques P Skorupskaite K Rozario KS Anderson RA George JT Feingold KR. Physiology of GnRH and gonadotropin secretion. In: Feingold KR Anawalt B Boyce A Chrousos G de Herder WW Dhatariya K Dungan K Hershman JM Hofland J Kalra S, editors. Endotext. South Dartmouth (MA: MDText.com, Inc. Copyright © 2000-2022, MDText.com, Inc (2000) 34.

[70] Goodman RL Herbison AE Lehman MN Navarro VM. Neuroendocrine control of gonadotropin-releasing hormone: Pulsatile and surge modes of secretion. J Neuroendocrinol (2022) 34:e13094. doi: 10.1111/jne.13094 35107859 PMC9948945

[71] Nagae M Uenoyama Y Okamoto S Tsuchida H Ikegami K Goto T. Direct evidence that KNDy neurons maintain gonadotropin pulses and folliculogenesis as the GnRH pulse generator. Proc Natl Acad Sci USA (2021) 118:e2009156118. doi: 10.1073/pnas.2009156118 33500349 PMC7865162

[72] Hrabovszky E Ciofi P Vida B Horvath MC Keller E Caraty A. The kisspeptin system of the human hypothalamus: sexual dimorphism and relationship with gonadotropin-releasing hormone and neurokinin b neurons. Eur J Neurosci (2010) 31:1984–98. doi: 10.1111/j.1460-9568.2010.07239.x 20529119

[73] Knoll JG Clay CM Bouma GJ Henion TR Schwarting GA Millar RP. Developmental profile and sexually dimorphic expression of kiss1 and kiss1r in the fetal mouse brain. Front Endocrinol (2013) 4:140. doi: 10.3389/fendo.2013.00140 PMC3795359 24130552

[74] Kauffman AS. Gonadal and nongonadal regulation of sex differences in hypothalamic Kiss1 neurones. J Neuroendocrinol (2010) 22:682–91. doi: 10.1111/j.1365-2826.2010.02030.x PMC3096441 20492362

[75] Cheng XB Jimenez M Desai R Middleton LJ Joseph SR Ning G. Characterizing the neuroendocrine and ovarian defects of androgen receptor-knockout female mice. Am J Physiol Endocrinol Metab (2013) 305:E717–726. doi: 10.1152/ajpendo.00263.2013 23880317

[76] Ruohonen ST Poutanen M Tena-Sempere M. Role of kisspeptins in the control of the hypothalamic-pituitary-ovarian axis: old dogmas and new challenges. Fertil Steril (2020) 114:465–74. doi: 10.1016/j.fertnstert.2020.06.038 32771258

[77] Clarkson J d'Anglemont de Tassigny X Moreno AS Colledge WH Herbison AE. Kisspeptin-GPR54 signaling is essential for preovulatory gonadotropin-releasing hormone neuron activation and the luteinizing hormone surge. J Neurosci (2008) 28:8691–7. doi: 10.1523/jneurosci.1775-08.2008 PMC6670827 18753370

[78] Novaira HJ Sonko ML Hoffman G Koo Y Ko C Wolfe A. Disrupted kisspeptin signaling in GnRH neurons leads to hypogonadotrophic hypogonadism. Mol Endocrinol (Baltimore Md.) (2014) 28:225–38. doi: 10.1210/me.2013-1319 PMC3896637 24422632

[79] Gutiérrez-Pascual E Martínez-Fuentes AJ Pinilla L Tena-Sempere M Malagón MM Castaño JP. Direct pituitary effects of kisspeptin: activation of gonadotrophs and somatotrophs and stimulation of luteinising hormone and growth hormone secretion. J Neuroendocrinol (2007) 19:521–30. doi: 10.1111/j.1365-2826.2007.01558.x 17532794

[80] Witham EA Meadows JD Hoffmann HM Shojaei S Coss D Kauffman AS. Kisspeptin regulates gonadotropin genes *via* immediate early gene induction in pituitary gonadotropes. Mol Endocrinol (Baltimore Md.) (2013) 27:1283–94. doi: 10.1210/me.2012-1405 PMC3725344 23770611

[81] Ma Y Awe O Radovick S Yang X Divall S Wolfe A. Lower FSH with normal fertility in Male mice lacking gonadotroph kisspeptin receptor. Front Physiol (2022) 13:868593. doi: 10.3389/fphys.2022.868593 35557961 PMC9089166

[82] Song WJ Mondal P Wolfe A Alonso LC Stamateris R Ong BW. Glucagon regulates hepatic kisspeptin to impair insulin secretion. Cell Metab (2014) 19:667–81. doi: 10.1016/j.cmet.2014.03.005 PMC4058888 24703698

[83] Hauge-Evans AC Richardson CC Milne HM Christie MR Persaud SJ Jones PM. A role for kisspeptin in islet function. Diabetologia (2006) 49:2131–5. doi: 10.1007/s00125-006-0343-z 16826407

[84] Bowe JE Foot VL Amiel SA Huang GC Lamb MW Lakey J. GPR54 peptide agonists stimulate insulin secretion from murine, porcine and human islets. Islets (2012) 4:20–3. doi: 10.4161/isl.18261 22192948

[85] Katugampola H King PJ Chatterjee S Meso M Duncan AJ Achermann JC. Kisspeptin is a novel regulator of human fetal adrenocortical development and function: A finding with important implications for the human fetoplacental unit. J Clin Endocrinol Metab (2017) 102:3349–59. doi: 10.1210/jc.2017-00763 PMC5587078 28911133

[86] Maguire JJ Kirby HR Mead EJ Kuc RE d'Anglemont de Tassigny X Colledge WH. Inotropic action of the puberty hormone kisspeptin in rat, mouse and human: cardiovascular distribution and characteristics of the kisspeptin receptor. PloS One (2011) 6:e27601. doi: 10.1371/journal.pone.0027601 22132116 PMC3222648

[87] León S Fernandois D Sull A Sull J Calder M Hayashi K. Beyond the brain-peripheral kisspeptin signaling is essential for promoting endometrial gland development and function. Sci Rep (2016) 6:29073. doi: 10.1038/srep29073 27364226 PMC4929565

[88] Radovick S Babwah AV. Regulation of pregnancy: Evidence for major roles by the uterine and placental Kisspeptin/KISS1R signaling systems. Semin Reprod Med (2019) 37:182–90. doi: 10.1055/s-0039-3400966 31972863

[89] Schaefer J Vilos AG Vilos GA Bhattacharya M Babwah AV. Uterine kisspeptin receptor critically regulates epithelial estrogen receptor α transcriptional activity at the time of embryo implantation in a mouse model. Mol Hum Reprod (2021) 27:gaab060. doi: 10.1093/molehr/gaab060 34524460 PMC8786495

[90] Matsui H Takatsu Y Kumano S Matsumoto H Ohtaki T. Peripheral administration of metastin induces marked gonadotropin release and ovulation in the rat. Biochem Biophys Res Commun (2004) 320:383–8. doi: 10.1016/j.bbrc.2004.05.185 15219839

[91] Thompson EL Patterson M Murphy KG Smith KL Dhillo WS Todd JF. Central and peripheral administration of kisspeptin-10 stimulates the hypothalamic-pituitary-gonadal axis. J Neuroendocrinol (2004) 16:850–8. doi: 10.1111/j.1365-2826.2004.01240.x 15500545

[92] Navarro VM Castellano JM Fernaíndez-Fernaíndez R Tovar S Roa J Mayen A. Characterization of the potent luteinizing hormone-releasing activity of KiSS-1 peptide, the natural ligand of GPR54. Endocrinology (2005) 146:156–63. doi: 10.1210/en.2004-0836 15375028

[93] Uenoyama Y Pheng V Tsukamura H Maeda KI. The roles of kisspeptin revisited: inside and outside the hypothalamus. J Reprod Dev (2016) 62:537–45. doi: 10.1262/jrd.2016-083 PMC5177970 27478063

[94] Jiang JH He Z Peng YL Jin WD Wang Z Han RW. Kisspeptin-13 enhances memory and mitigates memory impairment induced by Aβ1-42 in mice novel object and object location recognition tasks. Neurobiol Learn Mem (2015) 123:187–95. doi: 10.1016/j.nlm.2015.05.010 26103138

[95] Delmas S Porteous R Bergin DH Herbison AE. Altered aspects of anxiety-related behavior in kisspeptin receptor-deleted male mice. Sci Rep (2018) 8:2794. doi: 10.1038/s41598-018-21042-4 29434234 PMC5809376

[96] Qiu J Rivera HM Bosch MA Padilla SL Stincic TL Palmiter RD. Estrogenic-dependent glutamatergic neurotransmission from kisspeptin neurons governs feeding circuits in females. Elife (2018) 7:e35656. doi: 10.7554/eLife.35656 30079889 PMC6103748

[97] Wolfe A Hussain MA. The emerging role(s) for kisspeptin in metabolism in mammals. Front Endocrinol (2018) 9:184. doi: 10.3389/fendo.2018.00184 PMC5928256 29740399

[98] Harter CJL Kavanagh GS Smith JT. The role of kisspeptin neurons in reproduction and metabolism. J Endocrinol (2018) 238:R173–83. doi: 10.1530/joe-18-0108 30042117

[99] Izzi-Engbeaya C Dhillo WS. Emerging roles for kisspeptin in metabolism. J Physiol (2021) 600:1079–88. doi: 10.1113/jp281712 33977536

[100] Tolson KP Garcia C Yen S Simonds S Stefanidis A Lawrence A. Impaired kisspeptin signaling decreases metabolism and promotes glucose intolerance and obesity. J Clin Invest (2014) 124:3075–9. doi: 10.1172/jci71075 PMC4071390 24937427

[101] Sharma A Thaventhiran T Minhas S Dhillo WS Jayasena CN. Kisspeptin and testicular function-is it necessary? Int J Mol Sci (2020) 21:2958. doi: 10.3390/ijms21082958 PMC7216047 32331420

[102] Hsu MC Wang JY Lee YJ Jong DS Tsui KH Chiu CH. Kisspeptin modulates fertilization capacity of mouse spermatozoa. Reprod (Cambridge England) (2014) 147:835–45. doi: 10.1530/rep-13-0368 24567427

[103] Laoharatchatathanin T Terashima R Yonezawa T Kurusu S Kawaminami M. Augmentation of Metastin/Kisspeptin mRNA expression by the proestrous luteinizing hormone surge in granulosa cells of rats: Implications for luteinization. Biol Reprod (2015) 93:15. doi: 10.1095/biolreprod.115.127902 25995272

[104] Khristi V Chakravarthi VP Singh P Ghosh S Pramanik A Ratri A. ESR2 regulates granulosa cell genes essential for follicle maturation and ovulation. Mol Cell Endocrinol (2018) 474:214–26. doi: 10.1016/j.mce.2018.03.012 29580824

[105] Uenoyama Y Tomikawa J Inoue N Goto T Minabe S Ieda N. Molecular and epigenetic mechanism regulating hypothalamic Kiss1 gene expression in mammals. Neuroendocrinology (2016) 103:640–9. doi: 10.1159/000445207 26964105

[106] Lee EB Chakravarthi VP Wolfe MW Rumi MAK. ERβ regulation of gonadotropin responses during folliculogenesis. Int J Mol Sci (2021) 22:10348. doi: 10.3390/ijms221910348 34638689 PMC8508937

[107] Wang B Mechaly AS Somoza GM. Overview and new insights into the diversity, evolution, role, and regulation of kisspeptins and their receptors in teleost fish. Front Endocrinol (2022) 13:862614. doi: 10.3389/fendo.2022.862614 PMC8982144 35392133

[108] Li W Hu J Sun C Dong J Liu Z Yuan J. Characterization of kiss2/kissr2 system in largemouth bass (Micropterus salmoides) and Kiss2-10 peptide regulation of the hypothalamic-pituitary-gonadal axis. Comp Biochem Physiol B Biochem Mol Biol (2022) 257:110671. doi: 10.1016/j.cbpb.2021.110671 34450276

[109] Xiao Y Ni Y Huang Y Wu J Grossmann R Zhao R. Effects of kisspeptin-10 on progesterone secretion in cultured chicken ovarian granulosa cells from preovulatory (F1-F3) follicles. Peptides (2011) 32:2091–7. doi: 10.1016/j.peptides.2011.09.001 21924307

[110] Merhi Z Thornton K Bonney E Cipolla MJ Charron MJ Buyuk E. Ovarian kisspeptin expression is related to age and to monocyte chemoattractant protein-1. J assisted Reprod Genet (2016) 33:535–43. doi: 10.1007/s10815-016-0672-x PMC4818642 26879207

[111] Shahed A Young KA. Differential ovarian expression of KiSS-1 and GPR-54 during the estrous cycle and photoperiod induced recrudescence in Siberian hamsters (Phodopus sungorus). Mol Reprod Dev (2009) 76:444–52. doi: 10.1002/mrd.20972 PMC2660602 18937338

[112] Tanyapanyachon P Amelkina O Chatdarong K. The expression of kisspeptin and its receptor in the domestic cat ovary and uterus in different stages of the ovarian cycle. Theriogenology (2018) 117:40–8. doi: 10.1016/j.theriogenology.2018.05.019 29843081

[113] Cielesh ME McGrath BM Scott CJ Norman ST Stephen CP. The localization of kisspeptin and kisspeptin receptor in the canine ovary during different stages of the reproductive cycle. Reprod Domest Anim (2017) 52 Suppl 2:24–8. doi: 10.1111/rda.12841 27774658

[114] Basini G Grasselli F Bussolati S Ciccimarra R Maranesi M Bufalari A. Presence and function of kisspeptin/KISS1R system in swine ovarian follicles. Theriogenology (2018) 115:1–8. doi: 10.1016/j.theriogenology.2018.04.006 29698886

[115] Mishra GK Patra MK Singh LK Upmanyu V Chakravarti S M K. Kiss1 and its receptor: molecular characterization and immunolocalization in the hypothalamus and corpus luteum of the buffalo. Anim Biotechnol (2019) 30:342–51. doi: 10.1080/10495398.2018.1520715 30444171

[116] Gaytan F Gaytan M Castellano JM Romero M Roa J Aparicio B. KiSS-1 in the mammalian ovary: distribution of kisspeptin in human and marmoset and alterations in KiSS-1 mRNA levels in a rat model of ovulatory dysfunction. Am J Physiol Endocrinol Metab (2009) 296:E520–31. doi: 10.1152/ajpendo.90895.2008 19141682

[117] Ricu MA Ramirez VD Paredes AH Lara HE. Evidence for a celiac ganglion-ovarian kisspeptin neural network in the rat: intraovarian anti-kisspeptin delays vaginal opening and alters estrous cyclicity. Endocrinology (2012) 153:4966–77. doi: 10.1210/en.2012-1279 22869347

[118] Chakravarthi VP Ghosh S Dai E Pathak D Rumi MK. Transcriptome datasets of ESR2-regulated genes in rat granulosa cells during gonadotropin-induced follicle maturation. Data Brief (2020) 30:105405. doi: 10.1016/j.dib.2020.105405 32280735 PMC7139164

[119] Liu W Xin Q Wang X Wang S Wang H Zhang W. Estrogen receptors in granulosa cells govern meiotic resumption of pre-ovulatory oocytes in mammals. Cell Death Dis (2017) 8:e2662. doi: 10.1038/cddis.2017.82 28277543 PMC5386574

[120] Gal A Lin PC Cacioppo JA Hannon PR Mahoney MM Wolfe A. Loss of fertility in the absence of progesterone receptor expression in kisspeptin neurons of female mice. PloS One (2016) 11:e0159534. doi: 10.1371/journal.pone.0159534 27441639 PMC4956300

[121] Retana-Márquez S Juárez-Rojas L Ávila-Quintero A Rojas-Maya S Perera G Casillas F. Neuroendocrine disruption is associated to infertility in chronically stressed female rats. Reprod Biol (2020) 20:474–83. doi: 10.1016/j.repbio.2020.07.011 32807716

[122] Anuradha Krishna A. Kisspeptin regulates ovarian steroidogenesis during delayed embryonic development in the fruit bat, cynopterus sphinx. Mol Reprod Dev (2017) 84:1155–67. doi: 10.1002/mrd.22876 28804932

[123] Kirilov M Clarkson J Liu X Roa J Campos P Porteous R. Dependence of fertility on kisspeptin-Gpr54 signaling at the GnRH neuron. Nat Commun (2013) 4:2492. doi: 10.1038/ncomms3492 24051579

[124] León S Barroso A Vázquez MJ García-Galiano D Manfredi-Lozano M Ruiz-Pino F. Direct actions of kisspeptins on GnRH neurons permit attainment of fertility but are insufficient to fully preserve gonadotropic axis activity. Sci Rep (2016) 6:19206. doi: 10.1038/srep19206 26755241 PMC4709743

[125] Jayes FL Burns KA Rodriguez KF Kissling GE Korach KS. The naturally occurring luteinizing hormone surge is diminished in mice lacking estrogen receptor beta in the ovary. Biol Reprod (2014) 90:24. doi: 10.1095/biolreprod.113.113316 24337314 PMC4076404

[126] Pineda R Garcia-Galiano D Roseweir A Romero M Sanchez-Garrido MA Ruiz-Pino F. Critical roles of kisspeptins in female puberty and preovulatory gonadotropin surges as revealed by a novel antagonist. Endocrinology (2010) 151:722–30. doi: 10.1210/en.2009-0803 19952274

[127] Chakravarthi VP Ratri A Masumi S Borosha S Ghosh S Christenson LK. Granulosa cell genes that regulate ovarian follicle development beyond the antral stage: The role of estrogen receptor β. Mol Cell Endocrinol (2021) 528:111212. doi: 10.1016/j.mce.2021.111212 33676987 PMC8916094

[128] Lara H Dissen G Leyton V Paredes A Fuenzalida H Fiedler J. An increased intraovarian synthesis of nerve growth factor and its low affinity receptor is a principal component of steroid-induced polycystic ovary in the rat 1. Endocrinology (2000) 141:1059–72. doi: 10.1210/en.141.3.1059 10698182

[129] Convery M McCarthy GF Brawer JR. Remission of the polycystic ovarian condition (PCO) in the rat following hemiovariectomy. Anat Rec (1990) 226:328–36. doi: 10.1002/ar.1092260309 2327604

[130] Brawer J Richard M Farookhi R. Pattern of human chorionic gonadotropin binding in the polycystic ovary. Am J Obstet Gynecol (1989) 161:474–80. doi: 10.1016/0002-9378(89)90544-9 2764064

[131] Rehman R Zafar A Ali AA Baig M Alam F. Impact of serum and follicular fluid kisspeptin and estradiol on oocyte maturity and endometrial thickness among unexplained infertile females during ICSI. PLoS One (2020) 15:e0239142. doi: 10.1371/journal.pone.0239142 33112855 PMC7593084

[132] Navarro VM Fernández-Fernández R Castellano JM Roa J Mayen A Barreiro ML. Advanced vaginal opening and precocious activation of the reproductive axis by KiSS-1 peptide, the endogenous ligand of GPR54. J Physiol (2004) 561:379–86. doi: 10.1113/jphysiol.2004.072298 PMC1665361 15486019

[133] Hashizume T Saito H Sawada T Yaegashi T Ezzat AA Sawai K. Characteristics of stimulation of gonadotropin secretion by kisspeptin-10 in female goats. Anim Reprod Sci (2010) 118:37–41. doi: 10.1016/j.anireprosci.2009.05.017 19574004

[134] Kadokawa H Matsui M Hayashi K Matsunaga N Kawashima C Shimizu T. Peripheral administration of kisspeptin-10 increases plasma concentrations of GH as well as LH in prepubertal Holstein heifers. J Endocrinol (2008) 196:331–4. doi: 10.1677/joe-07-0504 18252956

[135] Jayasena CN Nijher GMK Comninos AN Abbara A Januszewki A Vaal ML. The effects of kisspeptin-10 on reproductive hormone release show sexual dimorphism in humans. J Clin Endocrinol Metab (2011) 96:E1963–72. doi: 10.1210/jc.2011-1408 PMC3232613 21976724

[136] Dorfman MD Garcia-Rudaz C Alderman Z Kerr B Lomniczi A Dissen GA. Loss of Ntrk2/Kiss1r signaling in oocytes causes premature ovarian failure. Endocrinology (2014) 155:3098–111. doi: 10.1210/en.2014-1111 PMC4097998 24877631

[137] Iwata K Kunimura Y Ozawa H. Hypothalamic kisspeptin expression in hyperandrogenic female rats and aging rats. Acta Histochem Cytochem (2019) 52:85–91. doi: 10.1267/ahc.19013 31777408 PMC6872488

[138] Fernandois D Na E Cuevas F Cruz G Lara HE Paredes AH. Kisspeptin is involved in ovarian follicular development during aging in rats. J Endocrinol (2016) 228:161–70. doi: 10.1530/joe-15-0429 26698566

[139] Abbara A Clarke S Islam R Prague JK Comninos AN Narayanaswamy S. A second dose of kisspeptin-54 improves oocyte maturation in women at high risk of ovarian hyperstimulation syndrome: a phase 2 randomized controlled trial. Hum Reprod (Oxford England) (2017) 32:1915–24. doi: 10.1093/humrep/dex253 PMC5850304 28854728

[140] Kasum M Franulic D Cehic E Oreskovic S Lila A Ejubovic E. Kisspeptin as a promising oocyte maturation trigger for *in vitro* fertilisation in humans. Gynecol Endocrinol (2017) 33:583–7. doi: 10.1080/09513590.2017.1309019 28393578

[141] Owens LA Abbara A Lerner A O'Floinn S Christopoulos G Khanjani S. The direct and indirect effects of kisspeptin-54 on granulosa lutein cell function. Hum Reprod (Oxford England) (2018) 33:292–302. doi: 10.1093/humrep/dex357 29206944

[142] Hunjan T Abbara A. Clinical translational studies of kisspeptin and neurokinin b. Semin Reprod Med (2019) 37:119–24. doi: 10.1055/s-0039-3400240 31869839

[143] Birinci H Vatansever HS Yüncü M. Effect of kisspeptin-54 on ovarian levels of pigment epithelium-derived factor (PEDF) and vascular endothelial growth factor (VEGF) in an experimental model of ovarian hyperstimulation syndrome (OHSS). Reproduction Fertil Dev (2021) 33:799–809. doi: 10.1071/rd21140 34610858

[144] Zhai J Liu J Zhao S Zhao H Chen ZJ Du Y. Kisspeptin-10 inhibits OHSS by suppressing VEGF secretion. Reprod (Cambridge England) (2017) 154:355–62. doi: 10.1530/rep-17-0268 28676533

[145] d'Anglemont de Tassigny X Colledge WH. The role of kisspeptin signaling in reproduction. Physiol (Bethesda Md.) (2010) 25:207–17. doi: 10.1152/physiol.00009.2010 20699467

[146] Peng J Tang M Zhang BP Zhang P Zhong T Zong T. Kisspeptin stimulates progesterone secretion *via* the Erk1/2 mitogen-activated protein kinase signaling pathway in rat luteal cells. Fertil Steril (2013) 99:1436–43.e1431. doi: 10.1016/j.fertnstert.2012.12.008 23312234

[147] Timologou A Zafrakas M Grimbizis G Miliaras D Kotronis K Stamatopoulos P. Immunohistochemical expression pattern of metastasis suppressors KAI1 and KISS1 in endometriosis and normal endometrium. Eur J Obstet Gynecol Reprod Biol (2016) 199:110–5. doi: 10.1016/j.ejogrb.2016.02.004 26918694

[148] Jeon YE Lee KE Jung JA Yim SY Kim H Seo SK. Kisspeptin, leptin, and retinol-binding protein 4 in women with polycystic ovary syndrome. Gynecol Obstet Invest (2013) 75:268–74. doi: 10.1159/000350217 23571154

[149] de Assis Rodrigues NP Laganà AS Zaia V Vitagliano A Barbosa CP de Oliveira R. The role of kisspeptin levels in polycystic ovary syndrome: a systematic review and meta-analysis. Arch Gynecol Obstet (2019) 300:1423–34. doi: 10.1007/s00404-019-05307-5 31584133

[150] Guzelkas I Orbak Z Doneray H Ozturk N Sagsoz N. Serum kisspeptin, leptin, neuropeptide y, and neurokinin b levels in adolescents with polycystic ovary syndrome. J Pediatr Endocrinol Metab (2022) 35:481–7. doi: 10.1515/jpem-2021-0487 35170267

[151] Liu J Qu T Li Z Yu L Zhang S Yuan D. Serum kisspeptin levels in polycystic ovary syndrome: A meta-analysis. J Obstet Gynaecol Res (2021) 47:2157–65. doi: 10.1111/jog.14767 33765692

[152] Tang R Ding X Zhu J. Kisspeptin and polycystic ovary syndrome. Front Endocrinol (2019) 10:298. doi: 10.3389/fendo.2019.00298 PMC6530435 31156550

[153] Lebbe M Woodruff TK. Involvement of androgens in ovarian health and disease. Mol Hum Reprod (2013) 19:828–37. doi: 10.1093/molehr/gat065 PMC3843026 24026057

[154] Romero-Ruiz A Skorupskaite K Gaytan F Torres E Perdices-Lopez C Mannaerts BM. Kisspeptin treatment induces gonadotropic responses and rescues ovulation in a subset of preclinical models and women with polycystic ovary syndrome. Hum Reprod (Oxford England) (2019) 34:2495–512. doi: 10.1093/humrep/dez205 PMC6936723 31820802

[155] Singla R Gupta Y Khemani M Aggarwal S. Thyroid disorders and polycystic ovary syndrome: An emerging relationship. Indian J Endocrinol Metab (2015) 19:25–9. doi: 10.4103/2230-8210.146860 PMC4287775 25593822

[156] Petrikova J Lazurova I Dravecka I Vrbikova J Kozakova D Figurova J. The prevalence of non organ specific and thyroid autoimmunity in patients with polycystic ovary syndrome. BioMed Pap Med Fac Univ Palacky Olomouc Czech Repub (2015) 159:302–6. doi: 10.5507/bp.2014.062 25485530

[157] Santos BR Dos Anjos Cordeiro JM Santos LC de Oliveira LS Mendonça LD Santos EO. Maternal hypothyroidism reduces the expression of the kisspeptin/Kiss1r system in the maternal-fetal interface of rats. Reprod Biol (2022) 22:100615. doi: 10.1016/j.repbio.2022.100615 35180577

[158] Shim YS Lee HS Hwang JS. Genetic factors in precocious puberty. Clin Exp Pediatr (2022) 65:172–81. doi: 10.3345/cep.2021.00521 PMC8990949 34665958

[159] Clarke H Dhillo WS Jayasena CN. Comprehensive review on kisspeptin and its role in reproductive disorders. Endocrinol Metab (Seoul) (2015) 30:124–41. doi: 10.3803/EnM.2015.30.2.124 PMC4508256 26194072

[160] Abbara A Hunjan T Ho VNA Clarke SA Comninos AN Izzi-Engbeaya C. Endocrine requirements for oocyte maturation following hCG, GnRH agonist, and kisspeptin during IVF treatment. Front Endocrinol (2020) 11:537205. doi: 10.3389/fendo.2020.537205 PMC7573298 33123084

[161] Jayasena CN Abbara A Veldhuis JD Comninos AN Ratnasabapathy R De Silva A. Increasing LH pulsatility in women with hypothalamic amenorrhoea using intravenous infusion of kisspeptin-54. J Clin Endocrinol Metab (2014) 99:E953–961. doi: 10.1210/jc.2013-1569 PMC4207927 24517142

